# Heart echinococcus cyst as an incidental finding: early detection might be life-saving

**DOI:** 10.1186/1749-8090-5-124

**Published:** 2010-12-08

**Authors:** Grigorios Tsigkas, Konstantinos Chouchoulis, Efstratios Apostolakis, Christina Kalogeropoulou, Nikolaos Koutsogiannis, Dimitra Koumoundourou, Dimitrios Alexopoulos

**Affiliations:** 1 Department of Cardiology, Patras University School of Medicine, Patras, Greece; 2 Department of Cardiothoracic Surgery, Patras University School of Medicine, Patras, Greece; 3 Department of Radiology, Patras University School of Medicine, Patras, Greece; 4 Department of Pathology, Patras University School of Medicine, Patras, Greece

## Abstract

We present a 46-year-old female smoker who was admitted to the emergency department of our hospital due to cough with blood-tinged sputum for the last four days before admission. Using echocardiography and Multi-Detector Computed Tomography (MDCT) heart Echinococcosis was diagnosed. Echinococcosis is a severe health issue in some geographical regions of the world. Hydatid infection of the heart is rare and the clinical presentation is usually insidious but there is always the lethal hazard of cyst perforation. Early diagnosis and an integrated treatment strategy are crucial. The results of surgical treatment of heart echinococcosis are better than the conservative strategy only. Extraction of the cyst combined with chemotherapy peri or post operative aiming to decrease the recurrences, consists the lege artis method of encountering this medical entity. Surgical excision was performed and the patient had an uneventful recovery and follow up at six and twelve months.

## Background

Hydatid disease is a common health problem in Mediterranean and especially in sheep-farming countries, caused by infection with the metacestode stage of the tapeworm Echinococcus. The liver is the most frequent primary site of Echinococcus granulosus infection in humans. Cardiac involvement is rare, and early diagnosis and prompt surgical intervention are critical, with complete resection of the germinal layer being of major importance for recurrence avoidance. Perioperative administration of albendazole has been reported to improve surgical outcome.

## Case presentation

**Figure 1:**
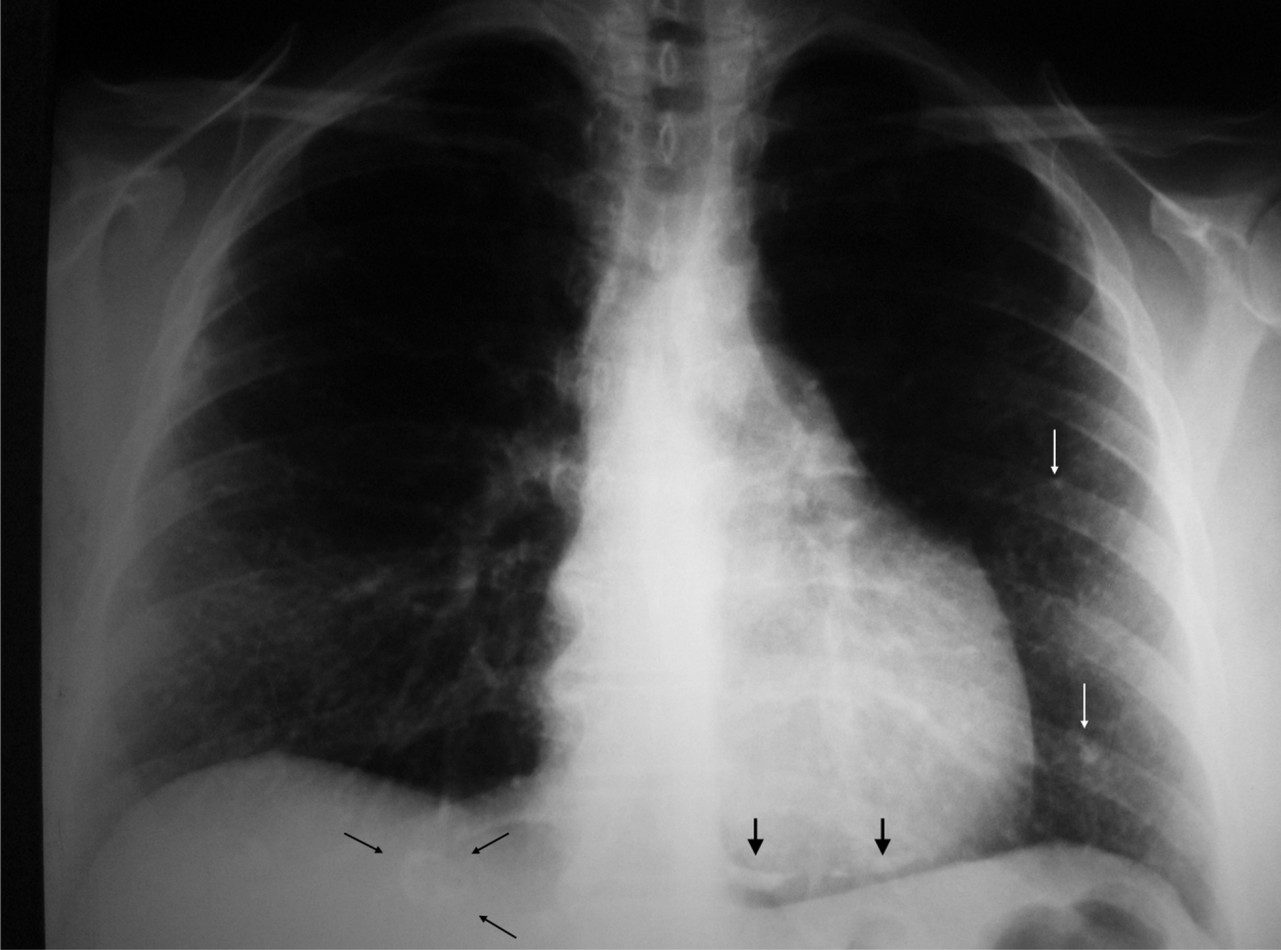
**Chest X-ray**. Tiny calcified nodules are visible on lung fields (white arrows). A coarse calcification of the heart cyst is projected at the heart base (thick black arrows). A calcified mass is also identified at the liver (thin black arrows).

**Figure 2:**
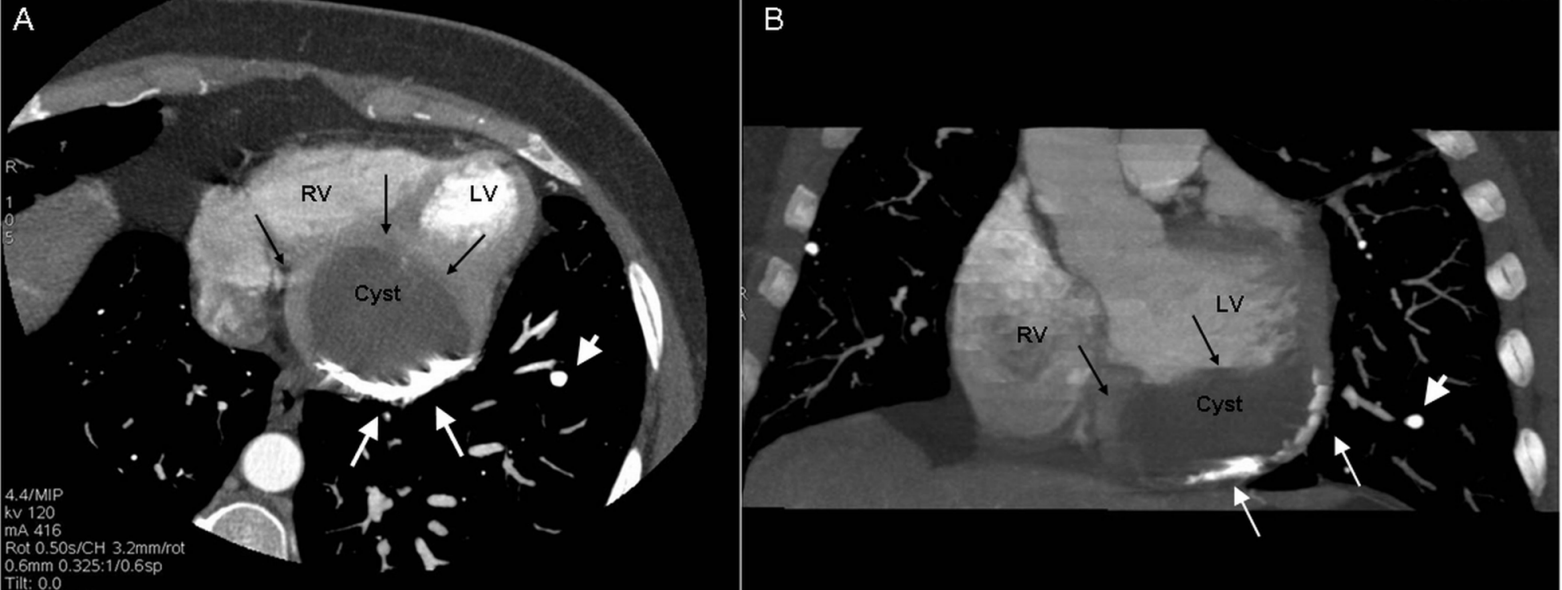
**MDCT**: Axial (Panel A) and coronal view (Panel B) of the heart reconstructed with thick slab MIP algorithm. A large unilocular cystic mass (black arrows) measuring 5 × 2 cm with partially calcified wall (white arrows), was found in the diaphragmatic surface (inferior aspect) of the heart. The mass was adjacent to the left ventricle compressing the inferior/posterior wall. Its outer contour was contiguous to the pericardium. Tiny calcified nodules are also noted in both lungs (white arrow heads).

**Figure 3:**
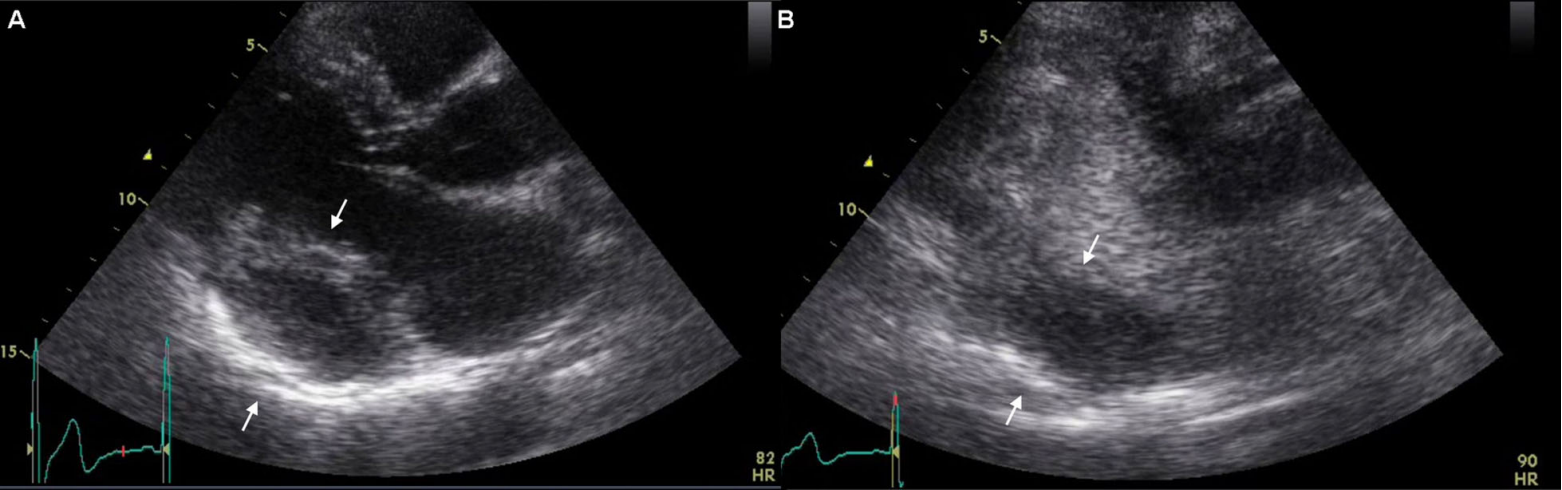
**Transthoracic echocardiography**. Panel A depicts long axis parasternal view of the hydatic cyst at the posterior wall of the heart (arrows). Panel B shows the same view using contrast media depicting the clear boarders of the cyst, without blood perfusion in the area of interest (arrows).

A 46-year-old female smoker was admitted to the emergency department of our hospital due to cough with blood-tinged sputum for the last four days before admission. She did not complaint of any other symptom. Her past medical history was unremarkable. She denied any exposure to toxic substances and gave no history of handling dogs or having ever reared sheep. The physical examination revealed no specific findings and there was no sign of respiratory or cardiovascular dysfunction. Lung auscultation revealed a mild decrease in the intensity of breath sounds, whereas heart examination was completely normal, without extra sounds or murmurs. The electrocardiogram (ECG) was lacking remarkable findings. The patient had negative reaction of the Mantoux tuberculin skin test. Moreover, all routine laboratory test results, including liver and kidney function tests, serum proteins and urinanalysis were normal. The erythrocyte sedimentation rate was normal (ESR 12 mm/hr). Sputum specimens for mycobacteria and other pathogens were smear and culture negative. Serological tests for cancer or virus were negative but serology for echinococcosis showed an indirect immunohemagglutination test positive. Other autoimmune markers were negative. Chest x-ray depicted multiple calcified cystic formations (Figure 1). A Multi-Detector Computed Tomography (MDCT) of the chest [1] and abdomen identified localized bronchiectatic lesions of the right upper lobe, calcified spots at both the lung fields, probably from previous disease (chickenpox, tuberculosis or occupational disease), a low density solitary lesion with peripheral calcification located at the myocardium of LV (compatible with calcified echinococcus cyst) (Figure 2) and other three similar but smaller findings were located at the liver. The CT scan of the brain showed no disease. No lung disease was diagnosed with the bronchoscopy. Transthoracic contrast echocardiography revealed a cyst-like echoluscent structure 5 × 2 cm, occupying the Posterior Wall of Left Ventricle (PWLV). It gave the impression of endomyocardial localization due to the decreased thickness of the PWLV (Figure 3). The rest of examination was normal with an estimated ejection fraction of 60%, without regional wall motion abnormalities. Coronary angiography revealed normal coronary arteries.

**Figure 4:**
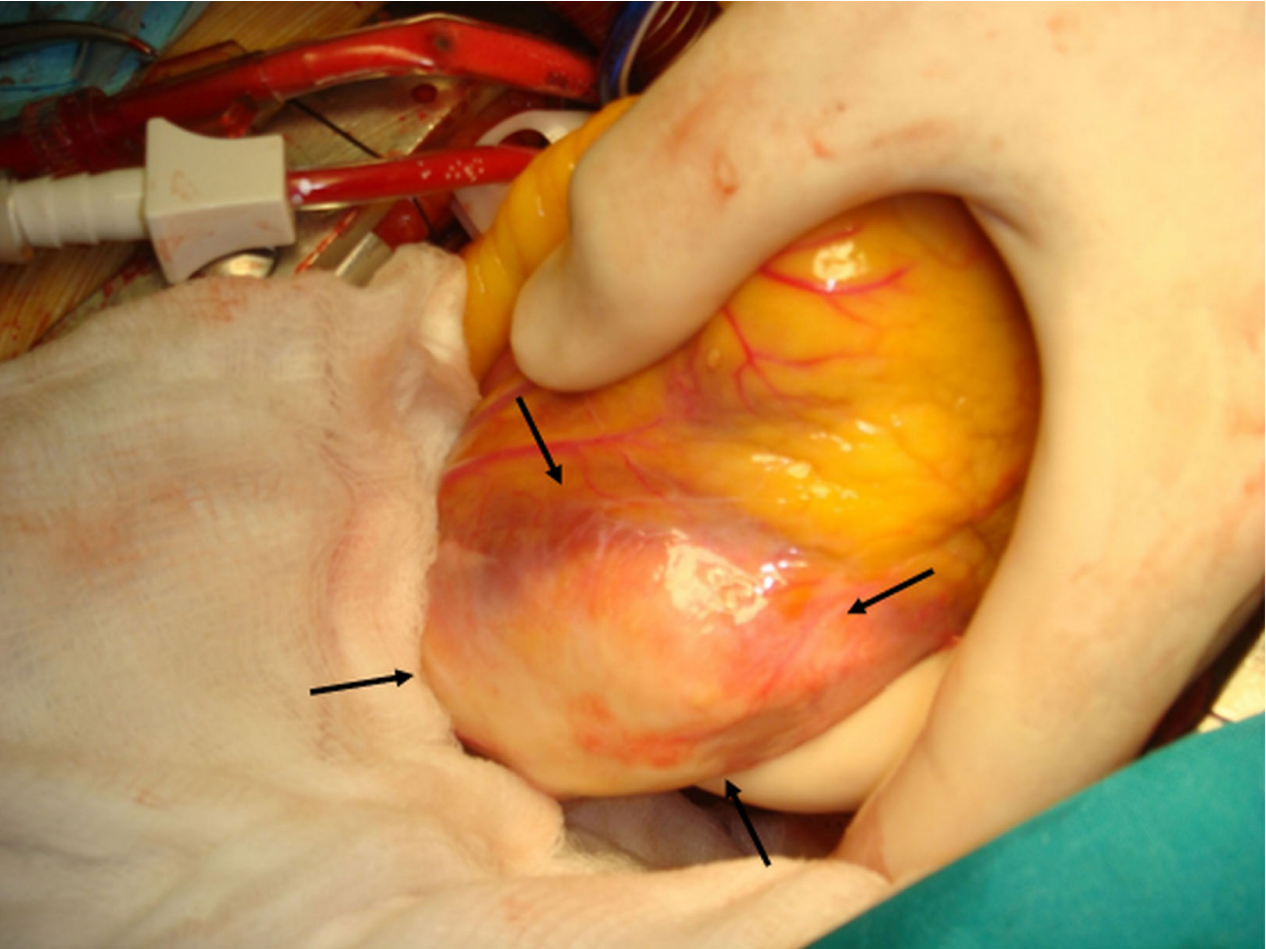
**Echinococcus cyst during operation**. The black arrows depict the cyst, after the elevation of the heart during operation.

**Figure 5:**
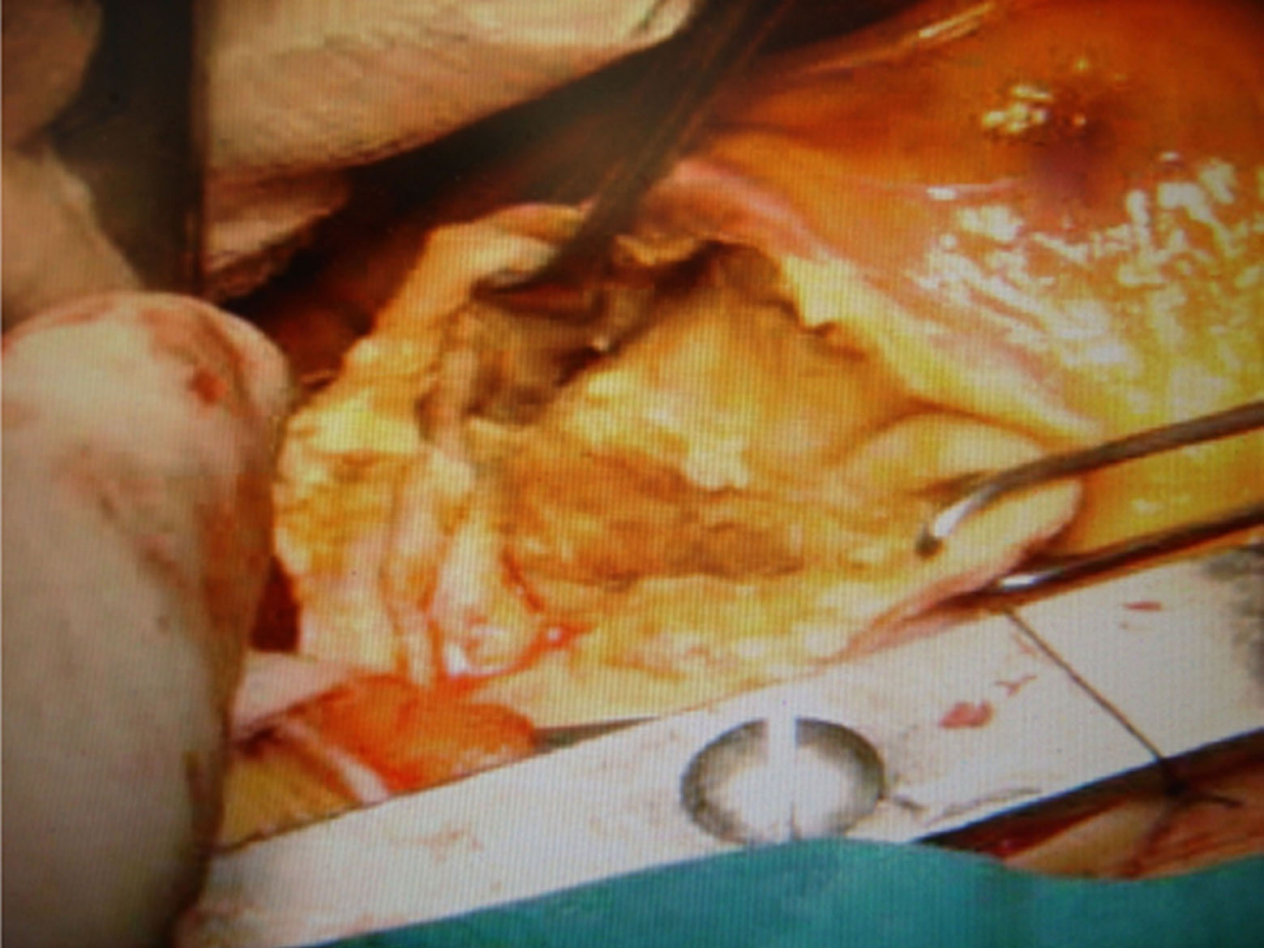
**The posterior wall of the cyst**, after the excision of sub-epicardial portion.

**Figure 6:**
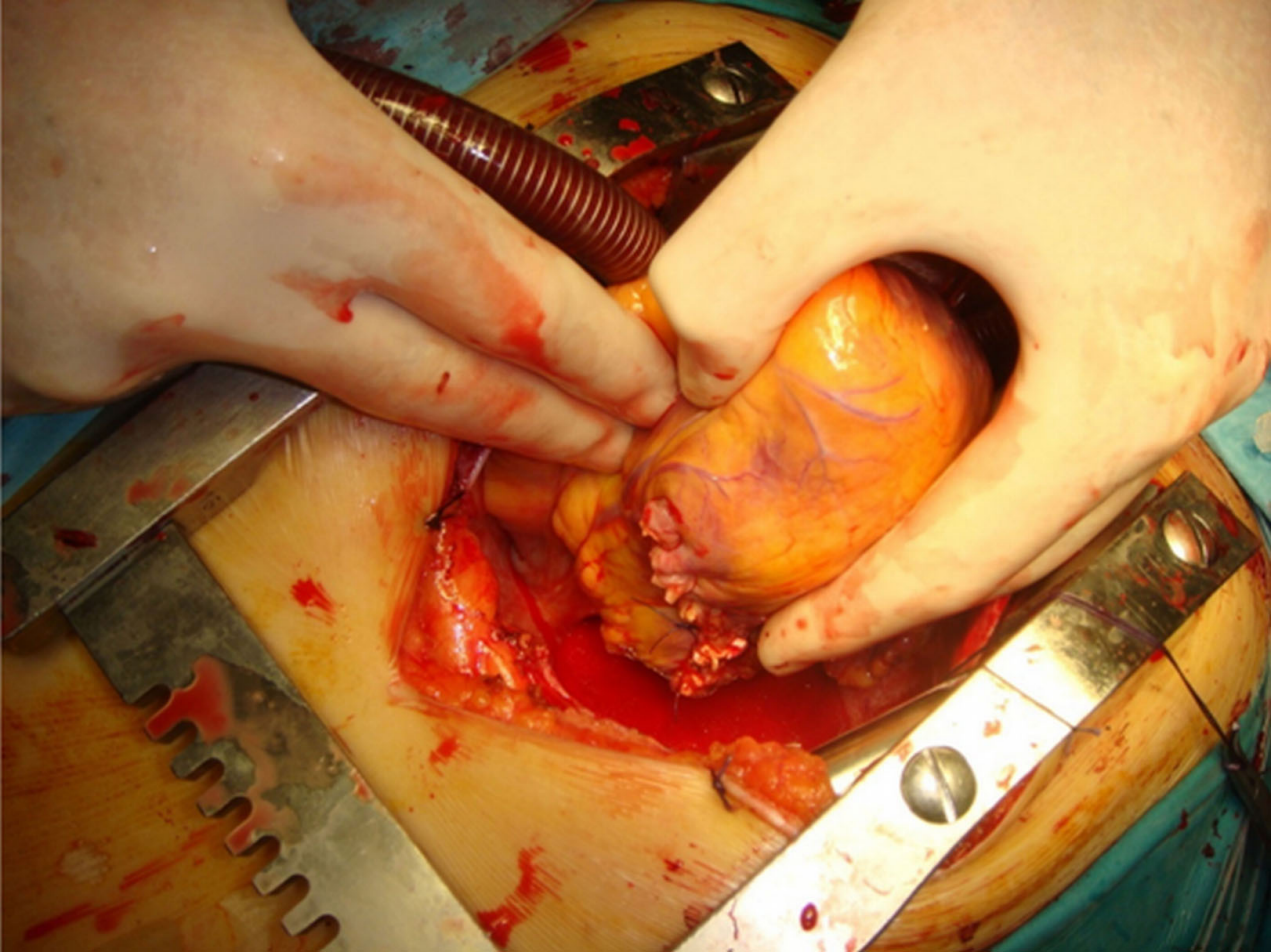
**The heart after the obliteration of the residual cavity**.

**Figure 7:**
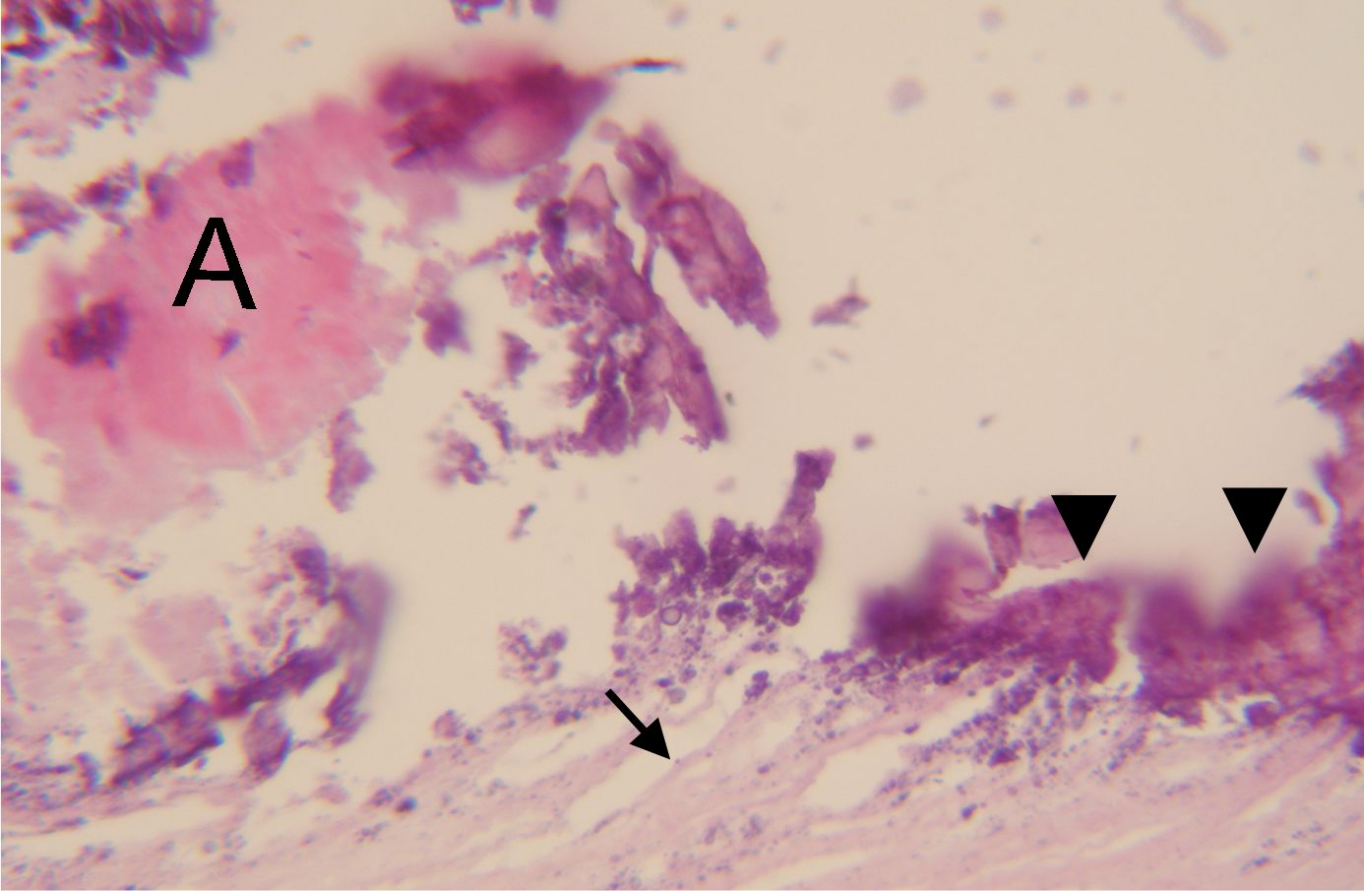
**Histologic examination of the cyst wall**. Microphotograph depicting the outer fibrous layer of the cyst (black arrow) and the presence of multiple calcifications (arrow heads) at the inner germinal layer. The letter (**A**) is marking an artifact.

Excision of the cardiac cyst was planned by using cardiopulmonary bypass and the patient received perioperative chemotherapy with albendazol 10 mg/kg. After a median sternotomy the patient was connected to the cardiopulmonary bypass by cannulating the ascending aorta and right atrium. The pericardial cavity was free of adhesions. Under condition of normothermic cardiopulmonary bypass the aorta was crossclamped and the heart was arrested with a dose of antegrade cold blood-based cardioplegia. The heart was lifted and the free surface of the cyst was seen as a white tense mass in the lateral wall of left ventricle **(**Figure 4**)**. By palpation, it was partially calcified. The lateral wall of the left ventricle was localized by gauzes irrigated by 10% NaCl solution. A small incision was made into the cyst corresponding its free wall, and a viscous sub-yellow fluid was aspirated. The sub-epicardial wall of the cyst was then excised and its contents were completely aspirated **(**Figure 5**)**. The cyst was then injected, first with 10 ml of 10% NaCl solution and then with 5 ml of povidone iodine solution, which was left inside for 3 min and then aspirated. The resultant cavity was then obliterated by a continuous 4-0 prolene suture **(**Figure 6**)**. To complete this obliteration, we avoided to use any prosthetic material such as pledgets, to prevent possible postoperative infection. After de-clamping of the aorta and de-airing of the heart, the patient was easily weaned off bypass with no inotropic support. Cytology of the aspirated cyst fluid and histology of the cyst wall was consistent with the diagnosis of hydatid cyst (Figure 7**)**. The patient had an uneventful recovery and at the fifth postoperative day she discharged from the hospital with a normal echocardiographic examination which confirmed preserved function of the mitral valve apparatus, with only minimal regurgitation.

**Figure 8:**
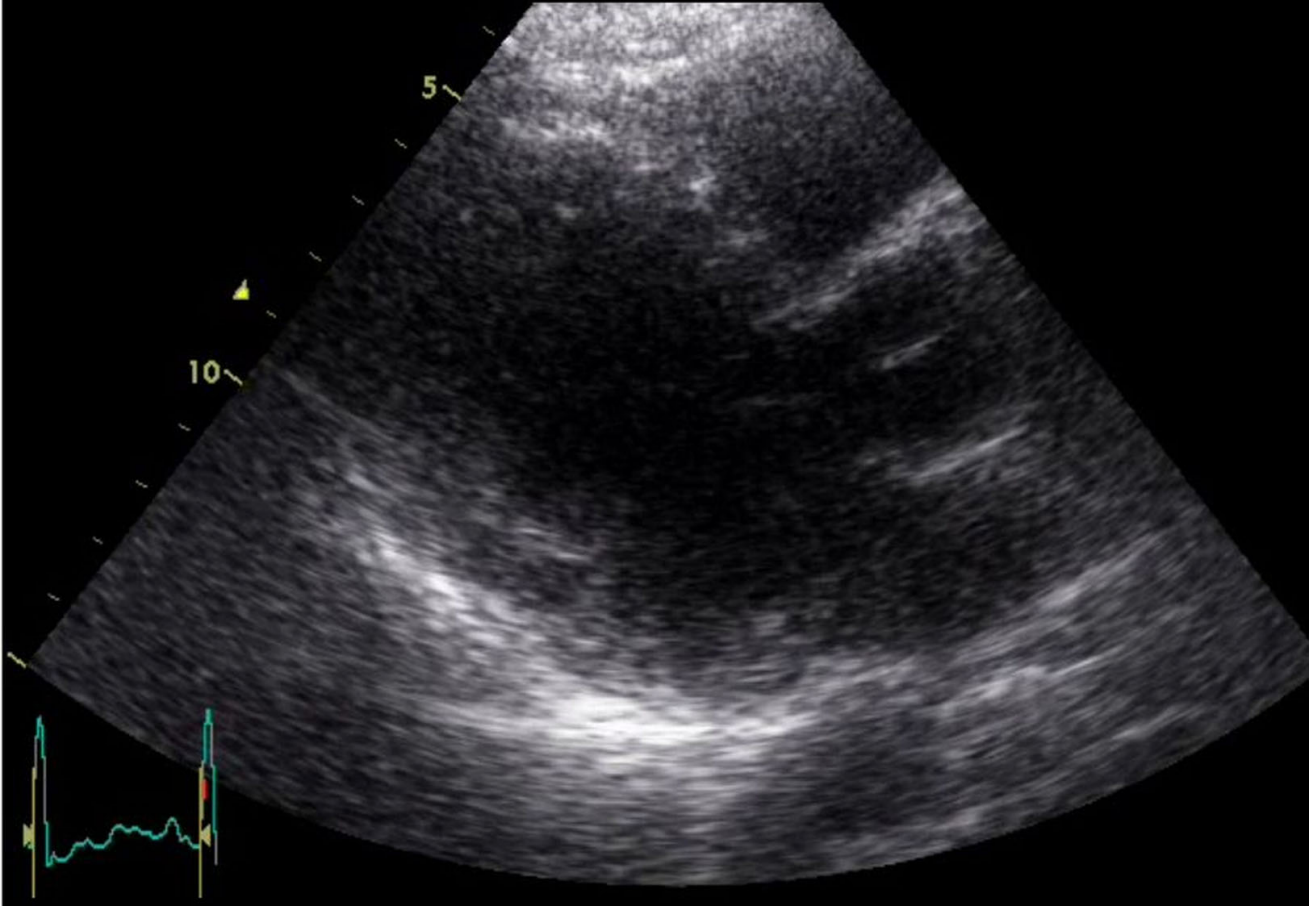
**Follow up transthoracic echocardiography**. Parasternal long axis view six months after the operation depicting a normal LV cavity.

Follow up at 6 and 12 months, with echocardiography (Figure 8) and CT scan, which confirmed the excellent post-surgery result without complications or recurrence of the disease. The findings of the liver were stable and brain CT was clear.

## Discussion

Echinococcosis was firstly described in the works of Hippocrates in the 4th century AD. Echinococcus granulosus is a cosmopolitan parasite. The overall incidence of Echinococcus infection is 0.4 per 100,000 persons. Humans are an accidental intermediary host, although most often found in the liver (60-70%) and lung (20-30%), hydatid cysts can occur in any organ or tissue. Involvement of the heart can occur from the systemic or pulmonary circulation or as direct extension from adjacent structures [2]. Isolated cardiac involvement is rare and occurs in only 0.02-2% of cases [3]. It can be located at any part of the heart and the manifestations depend on the size, location, and integrity of the cyst. The left ventricle myocardium is involved 2-3-fold more frequently than the right one with fewer cases at interventricular septum. Involvement of left and right atrium is approximately equal [4]. Pericardial cysts occur mostly in multifocal heart echinococcosis. Solitary pericardial cysts are rare [5]. Although the serologic reactions for hydatid cyst provide essential information, their sensitivity is not high and parameters frequently do not correspond to the morphological changes of the disease [6]. Transthoracic echocardiography and more recently, contrast echocardiography, computed tomography, and magnetic resonance imaging are the most important tools for diagnosis and follow up of the patient.

Cyst perforation is the most hazardous complication of heart echinococcosis. As a rule, left ventricle cysts perforate out of the cavity (10 to 20 times more frequently than right ventricle cysts), and right ventricle cysts perforate into it [7,8]. The frequency of intracardiac perforation is very high (25-40%). After cyst perforation 75% of the patients died from septic shock or embolic complications [9,10].

Whereas cysts in other organs may be treated both by chemotherapy and surgical manipulations, in the case of heart echinococcosis it is impossible to administer antihelmintic medicines prior to surgery due to the risk of cyst wall destruction and rupture. In addition the results of surgical treatment of heart echinococcosis are better than the conservative strategy only [11]. On the other hand, there have been described major surgical implications from rupture, with systemic or pulmonary embolization, pericardial dissemination, purulent inflammation, and sepsis [12,13].

## Conclusions

In view of the difficulties of the diagnosis and the progressive and dangerous complications in its natural course, surgical treatment of cardiac echinococcosis is urgent [14,15]. In conclusion, the treatment of heart echinococcosis should be a combination of surgical intervention with chemotherapy during or post-operative period aiming to decrease the recurrences.

## Consent

Written informed consent was obtained from the patient for publication of this case report and any accompanying images. A copy of the written consent is available for review by the Editor-in-Chief of this journal.

## Competing interests

The authors declare that they have no competing interests

## Authors' contributions

G.T. has made substantial contributions to conception and design, has been involved in drafting the manuscript and revising it critically for important intellectual content, K.C. has been involved in drafting the manuscript, E.A. has been involved in operating the patient and drafting the manuscript, C.K. has made substantial contributions to design the manuscript and has been involved in interpretation of CT, N.K. carried out the echocardiogram studies and has made substantial contributions of data analysis, D.K. carried out the histopathological analysis and interpretation and D.A. has made substantial contribution to design and has given the final approval of the version to be published. All authors read and approved the final manuscript.
